# Genetic engineering of primary human mesenchymal stromal cells using the CRISPR/Cas9 synergistic activation mediator

**DOI:** 10.1016/j.gendis.2026.102138

**Published:** 2026-03-17

**Authors:** M.C. Killer, X. Zhang, T. Adhikary, C. Kasper, A. Neubauer, M. Frech, C. Brendel

**Affiliations:** a Department of Hematology, Oncology and Immunology, Marburg University, Marburg 35041, Germany; b Department of Regenerative Medicine, Tongji University, Shanghai 200092, China; c Institute of Molecular Biology and Tumor Research, Marburg University, Marburg 35041, Germany; d Department for Biotechnology and Food Technology, BOKU University, Vienna 1180, Austria

Mesenchymal stromal cells (MSCs) have been tested in more than 1000 clinical trials for various allogeneic applications such as tissue replacement or immunosuppression in autoimmune disorders, and almost no adverse events have been reported so far. Therefore, enhancement of endogenous gene expression in MSCs from healthy donors via CRISPR/Cas technologies may become an alternative mode of cellular therapy.^1^ The CRISPR/Cas9 synergistic activation mediator (SAM), developed by Konermann et al, utilizes an engineered dead Cas9 (dCas9), which is not capable of cleaving target DNA sequences and does not introduce DNA strand breaks.^2^ To date, only a few studies report the usage of CRISPR/Cas9 in immortalized (iMSCs) or primary MSCs (pMSCs). CRISPR/dCas9-driven induction of transcriptional regulators of adipogenic differentiation had been described in an iMSC cell line.^3^ We sought to engineer MSCs to express the non-constitutive class II major histocompatibility complex transactivator (*CIITA*), the co-activator of human leukocyte antigen (HLA) class II genes (Fig. 1A). Different optimization approaches for gene induction in adult human iMSCs and pMSCs were compared and described (see Supplementary materials and methods), and some were found to be unsuccessful (Table S1). The T47D and HEK-293T cell lines served as positive controls and were applied in parallel to pMSCs. At first, the successful genomic integration and translation of the SAM system itself were verified by PCR analysis of the *dCas9-VP64* and *MS2-P65-HSF1* sequences in gDNA from transduced pMSCs and by Western blotting analysis of protein samples from transfected HEK-293T and transduced pMSCs (Fig. S1). Next, the human CCAAT/enhancer binding protein alpha (*CEBPA)* gene, which is up-regulated by MSCs upon adipogenic differentiation, was chosen as the first target for SAM-mediated activation in pMSCs.^3^ Three different gRNAs targeting the promoter of *CEBPA* (gRNA_CEBPA) were initially tested regarding their efficiency by transient transfection of HEK-293T cells before transduction of T47D cells and pMSCs. The gRNA plasmids were combined with the plasmids encoding the dCas9-VP64 and MS2-p65-HSF1 fusion proteins. Upon quantitative reverse transcription PCR, the highest levels of *CEBPA* gene expression were observed with gRNA_CEBPA #1 and #3 in all cell lines. An additive effect could be observed with the combined application of all three gRNAs (Fig. S2). To activate the expression of *CIITA* and the downstream target gene *HLA-DR*, pMSCs and peripheral blood mononuclear cells (PBMCs) as a control were exposed to different doses of interferon gamma (IFN-γ). MSCs do not express HLA-class II antigens on their cell surface constitutively, but quantitative reverse transcription PCR and protein analysis revealed that expression levels of both *CIITA* and *HLA-DR* were enhanced in a dose- and time-dependent manner by IFN-γ (Fig. S3A, S3C, S3D). PBMCs were barely susceptible to activation of *CIITA* and *HLA-DR* (Fig. S3B), even though the basal expression levels of both genes were distinctly higher in PBMCs in direct comparison (Fig. S4).

**Figure 1:**
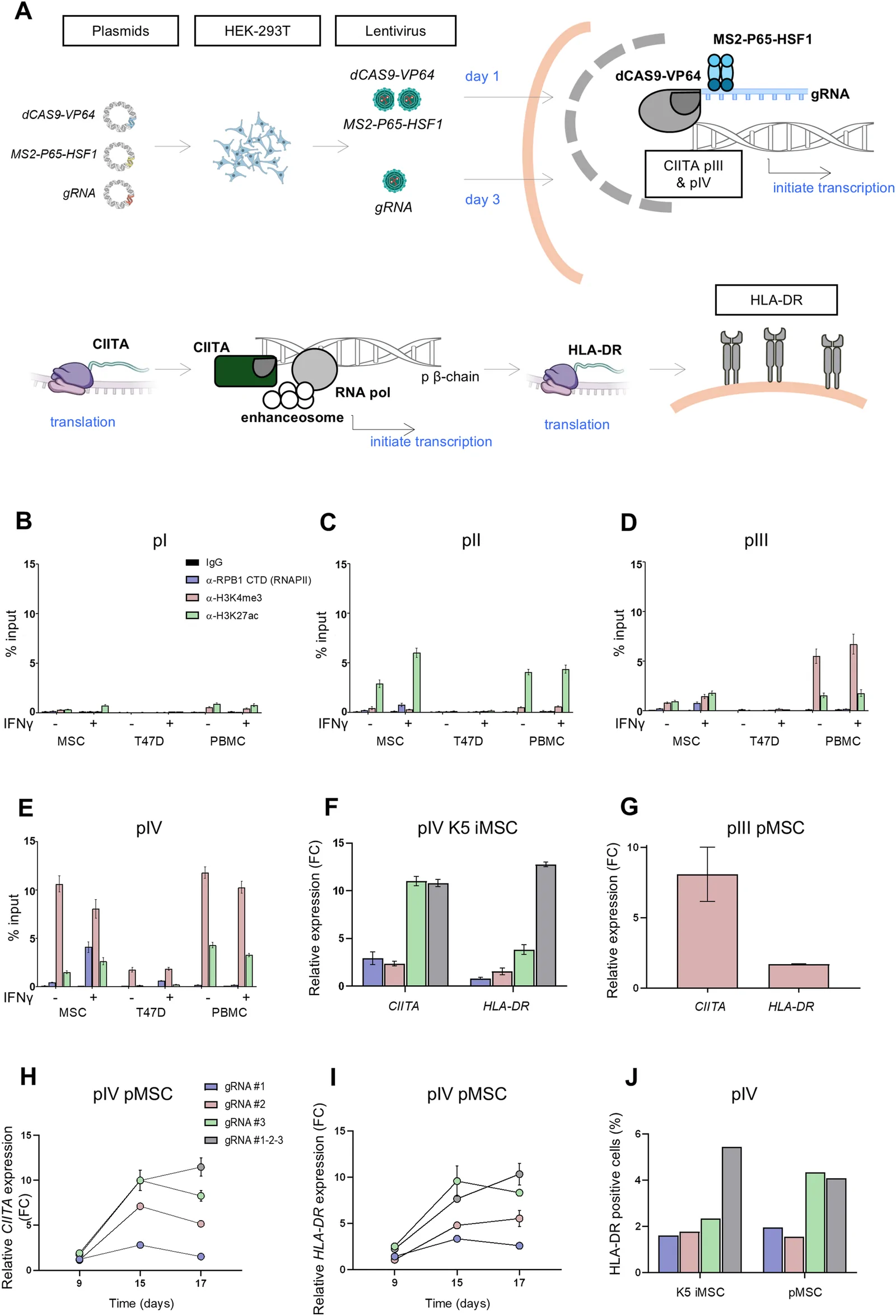
Strategy for genetic engineering of human primary mesenchymal stromal cells (pMSCs): Promotor selection and transduction efficacy. **(A)** Schematic overview of the workflow of the study for HLA-DR induction in pMSCs and control cells: pMSCs and control cells were transduced with the CRISPR/Cas9 SAM to express CIITA, which assembles a complex of molecules (enhanceosome) to activate RNA polymerase (RNA pol) at MHC class II promoter regions. After intracellular translation, assembly of alpha and beta chains, and glycosylation, HLA-DR molecules are expressed on the cellular surface. Icons for illustrations were taken from the NIH BioArt database (http://bioart.niaid.nih.gov). **(B**–**E)** Antibodies directed against RNA polymerase II (RNAPII), tri-methylation of the 4th lysine residue of histone H3 (H3K4me3), and acetylation of the 27th lysine residue of histone H3 (H3K27ac) were employed for chromatin immunoprecipitation (ChIP) in pMSCs, T47D cells, and peripheral blood mononuclear cells (PBMCs). Levels of gene transcription were measured via input-normalized quantitative PCR with primer pairs specifically targeting the four different promoter regions (pI–pIV) of *CIITA*. Cells were cultured with and without IFN-γ treatment (100 U/mL). Legend for (B–E) shown in (B). **(F**–**J)** CRISPR/Cas9 SAM-induced expression of *CIITA* and *HLA-DR* in human immortalized MSCs (iMSCs) and pMSCs. Expression analyses are depicted as fold change (FC). Legend for (F–J) is shown in (H).

To verify which of the four promoters (pI–pIV) of *CIITA* is activated in response to IFN-γ treatment in MSCs, a chromatin immunoprecipitation analysis was conducted as previously described.^4^ PBMCs, T47D cells, and pMSCs were treated with IFN-γ for 2 h, and subsequently, the chromatin immunoprecipitation analysis was performed with antibodies directed against RNA polymerase II (RNAPII), tri-methylation of the 4th lysine residue of histone H3 (H3K4me3), and acetylation of the 27th lysine residue of histone H3 (H3K27ac). For pI, none of the markers were detectable in any of the applied samples (Fig. 1B). At pII, the H3K27ac signal was predominantly induced in pMSCs under IFN-γ treatment but showed no induction by IFN-γ in PBMCs (Fig. 1C). In contrast, moderate enhancement of all three markers was detected at pIII in pMSCs after IFN-γ treatment. In PBMCs, H3K4me3 was induced (Fig. 1D). H3K4me3 signals were observed at pIV for all cell types, with or without IFN-γ treatment. In addition, H3K27ac signal induction was observed at pIV in pMSCs, and RNAPII binding at pIV was induced in both pMSCs and T47D cells after IFN-γ treatment (Fig. 1E). Thus, *CIITA* pIII and pIV were predominantly activated upon IFN-γ treatment in all cell types, and the strongest induction in pMSCs was observed for pIV. Consequently, *CIITA* promoters pIII and pIV were chosen as targets for SAM-mediated gene activation. At first, three different gRNA oligonucleotides for targeting each *CIITA* pIII (gRNA_CIITA-pIII) and pIV (gRNA_CIITA-pIV) were tested by transient transfection of HEK-293T cells, followed by transduction of T47D cells (Fig. S5). With gRNA_CIITA-pIII #2 and the pooled gRNAs, *CIITA* and *HLA-DR* expression was strongly induced in HEK-293T cells, while no enhancement was observed with gRNA_CIITA-pIII #1 and #3. Gene activation was also observed when applying gRNA_CIITA-pIII #2 in T47D cells. When testing the gRNAs targeting *CIITA* pIV, up-regulation of *CIITA* and *HLA-DR* was observed with gRNA_CIITA-pIV #2 and #3, and a pooled application of the three different gRNAs again exhibited an additive effect in both cell lines.

The protocol was subsequently applied to K5 iMSCs.^5^ Here, gRNA_CIITA-pIV #3 showed a strong effect on *CIITA* and *HLA-DR* gene expression, while the other gRNAs were less effective. The pooled application of all three gRNAs caused the highest activation of *HLA-DR* in iMSCs (Fig. 1F). Both *CIITA* pIII and pIV were tested for subsequent induction experiments in pMSCs. Although the H3K27ac signal in pMSCs was induced in the pIII promoter region under IFN-γ treatment, the induction of *CIITA* in pMSCs via pIII was rather difficult. With application of the transduction enhancer polybrene and spinofection, an 8-fold induction of *CIITA* gene expression was observed, and *HLA-DR* gene was also up-regulated (Fig. 1G), but spinofection was detrimental to long-term cell survival. To investigate whether an epigenetic inhibitor could facilitate accessibility of the promoter, valproic acid and decitabine were tested. Neither of the agents clearly enabled *CIITA* activation by gRNA_CIITA-pIII #2 (Fig. S7).

Last, *CIITA* pIV was tested for SAM-mediated target gene induction in pMSCs. Expression of *CIITA* and *HLA-DR* was analyzed at different time points. The gRNA pool increased *CIITA* expression to 11-fold after 17 days (Fig. 1H). Also, for *HLA-DR*, the strongest inducing effect was observed with the pooled gRNAs after 17 days (10-fold; Fig. 1I). Finally, both K5 iMSCs and pMSCs were evaluated for HLA-DR surface protein in a flow cytometric analysis after transduction. In K5 iMSCs, gRNA_CIITA-pIV #3 induced HLA-DR protein expression in 2.3% of cells, while the gRNA pool induced expression in 5.5% of cells. In pMSCs, gRNA_CIITA-pIV #3 and the gRNA pool similarly induced HLA-DR protein expression in 4.3% and 4.1% of cells, respectively (Fig. 1J).

Taken together, we report the proof of principle that lentiviral transduction of pMSCs with the CRISPR/dCas9 SAM system is capable of inducing genes and gene cascades that are not expressed constitutively. Efficacy of gene activation depends on optimized *in vitro* cell maintenance, gRNA capabilities, promoter recruitment, and design of the vector system. SAM-engineered human pMSCs are considered safe vehicles for activation and restoration of genes.^1^ With the protocol established in this study, we were able to achieve stable activation of *CIITA* and *HLA-DR* not only in immortalized but also in primary human MSCs. Our strategy holds the potential for MSC engineering for various applications in cell-based therapeutic strategies.

## CRediT authorship contribution statement

**M.C. Killer:** Writing – original draft, Visualization, Validation, Formal analysis, Data curation. **X. Zhang:** Supervision, Resources, Methodology, Conceptualization. **T. Adhikary:** Validation, Resources, Methodology, Formal analysis, Data curation, Conceptualization. **C. Kasper:** Resources, Methodology. **A. Neubauer:** Writing – review & editing, Funding acquisition. **M. Frech:** Supervision, Project administration, Methodology, Investigation, Formal analysis, Data curation, Conceptualization. **C. Brendel:** Writing – review & editing, Writing – original draft, Visualization, Validation, Supervision, Project administration, Methodology, Investigation, Funding acquisition, Data curation, Conceptualization.

## Ethics declaration

The local Ethics Committee of the Faculty of Medicine of the Philipps-University Marburg, Germany, approved the collection of human bone marrow from hip replacement surgery (Study 25-10; March 08, 2010; Title: Cultivation and expansion of human mesenchymal stem cells under GMP-compliant conditions). The donor gave written informed consent.

The Ethics Committee of the Faculty of Medicine of the Justus Liebig University Giessen, Germany, approved the use of remainders of donated blood, which cannot be used for blood transfusions, for research purposes (Study 05/00; November 30, 2000; Title: Research on residual blood intended to be discarded). Donors gave written informed consent.

The Ethics Committee of the Medical University of Vienna, Austria, approved the collection of adipose tissue for the establishment of the K5 immortalized MSC cell line with informed consent of a 65-year-old female donor who had undergone plastic surgery (EK Nr. 957/2011; January 30, 2013).^5^ The co-author C.K. provided K5 immortalized MSCs non-commercially under a Material Transfer Agreement.

## Funding

This study was funded by the Deutsche José Carreras Leukämie-Stiftung (No. DJCLS 12R/2017 and DJCLS 15R/2020 to C.B. and M.F.; No. DJCLS AH 06-01 to A.N.).

## Conflict of interests

The authors declared no conflict of interests related to this work.
