# Supplementary material for: Genetic engineering of primary human mesenchymal stromal cells using the CRISPR/Cas9 synergistic activation mediator
Source: Genes Dis. 2026 Mar 17;14(3):102138. doi: 10.1016/j.gendis.2026.102138 (PMC13718837; doi:10.1016/j.gendis.2026.102138)
Supplement: Supplementary file 1 [file mmc1.docx]

**Supplementary Information**

**SI Material and Methods**

**Isolation of primary MSC from human bone marrow**

Human bone marrow was aspirated from the iliac crest of one healthy donor. Expansion of pMSC was done as described before,^[1]^ and pMSC were stored in cell harvest bags in the second passage. For all experiments, second passage pMSC were employed in order to minimize the impact of cellular senescence. Cells were thawed, counted, expanded for one passage under standard culture conditions and re-frozen in cryotubes in liquid nitrogen. In the course of the study, individual tubes of cells were thawed freshly and allowed to equilibrate for 5 days before each experiment.

**Isolation of primary PBMC from buffy coats**

Isolation of primary peripheral blood mononuclear cells (PBMC) from buffy coats was done as follows: 20 ml buffy coat (University Hospital of Giessen and Marburg, transfusion medicine, Giessen, Germany) were diluted with 30 ml PBS, and 35 ml of the solution were layered on top of 15 ml Ficoll-Paque PLUS reagent (GE Healthcare Life Sciences, Chalfont St Giles, UK). Density gradient centrifugation was done for 20 min at 800 *g*. After centrifugation, PBMC contained in the middle layer were extracted using a transfer pipette and washed with PBS.

**Cultivation of established cell lines and primary cells**

HEK-293T cells were grown in DMEM with 4.5 g/l D-Glucose and T47D cells in RPMI 1640 medium (both Gibco by Thermo Fisher Scientific), both supplemented with 10% fetal calf serum (Capricorn Scientific, Ebsdorfergrund, Germany). Both pMSC and the immortalized MSC line K5 iMSC^[2]^ were grown in DMEM with 1 g/l D‑Glucose (Gibco) supplemented with 10% human platelet lysate (HPL; PL BioScience, Aachen, Germany). Heparin (PL BioScience) was added to a final concentration of 2 units/ml to prevent HPL coagulation. Primary PBMC were cultured in RPMI 1640 medium supplemented with 10% inactivated AB serum, 1% 4-(2-hydroxyethyl)-1-piperazineethanesulfonic acid (HEPES) buffer solution (1 M; Gibco), 1% MEM non-essential amino acid solution (100x; Sigma-Aldrich) and 1% sodium pyruvate (100 mM; Gibco).

1% Penicillin-Streptomycin (Sigma-Aldrich) was added to all media before use. All human cells were cultivated at 37 °C under hypoxic conditions through application of 5% CO_2_. Immortalized K5 iMSC were created and provided by C. Kasper and were maintained as described.^1^

Antibiotics used for cell selection were Zeocin (100 mg/ml), Hygromycin B Gold (100 mg/ml) and Blasticidin (10 mg/ml) (InvivoGen, San Diego, USA).

**Transfection assays**

For transfection, 3x10^5^ cells were seeded and plasmids were added with a calcium phosphate solution dropwise. Medium was changed after 14 - 16 h, and cells were grown until 3 days post transfection before being harvested for RNA isolation. For testing of gRNA efficiencies, equal amounts of lenti dCAS-VP64_Blast, lenti MS2-P65-HSF1_Hygro and the respective lenti sgRNA(MS2)_zeo + gRNA were combined. Different amounts of total plasmid DNA were tested. Moreover, HEK-293T cells were used for production of lentiviral particles. In that case, 3.5x10^6^ cells were seeded, and 2 µg pMD2.G, 5 µg psPAX2 and 7 µg lentiviral transfer plasmid were added. In case of calcium phosphate transfections with the PB-UniSAM plasmid,^[3]^ 3.5x10^6^ pMSC or HEK‑293T cells were seeded, and 15 µg PB‑UniSAM and 5 µg pCMV-hyPBase were added.

Transfection of HEK-293T cells for production of lentiviral particles was also done using Lipofectamine 3 000 (Thermo Fisher Scientific). Therefore, 3.5x10^6^ cells were seeded and the reagent was prepared and applied according to the manufacturer’s protocol. 2 µg pMD2.G, 5 µg psPAX2 and 7 µg lentiviral transfer plasmid were applied for each preparation. Cells were cultured for 3 days post transfection.

**Oligonucleotides and plasmids**

Guide RNAs (gRNAs), primers and plasmids used within this study are listed below. Target sequence specificity of all gRNAs and primers was verified using the Primer-BLAST online tool,^[4]^ and all oligonucleotides for this study were ordered from Eurofins MWG Operon (Ebersberg, Germany). The plasmids psPAX2 and pMD2.G were gifts from Didier Trono. The plasmids lenti dCAS-VP64_Blast, lenti MS2-P65-HSF1_Hygro and lenti sgRNA(MS2)_zeo backbone were gifts from Feng Zhang. The plasmid PB-UniSAM was a gift from Lesley Forrester.

**Molecular cloning of recombinant lentiviral transfer plasmids**

All gRNA oligonucleotides were designed to have matching overhangs for molecular cloning in BsmbI or BbsI digested plasmids after annealing. Respective forward and reverse oligonucleotides of each gRNA were annealed using the following mix: 1 µl of each oligonucleotide (100 µM), 1 µl T4 DNA Ligase Reaction Buffer (New England Biolabs, NEB), 0.5 µl T4 Polynucleotide Kinase (10 000 units/ml; NEB), 6.5 µl ddH_2_O. Annealing was done in a peqSTAR thermal cycler (Peqlab Biotechnologie, Erlangen, Germany) at 95 °C for 3 minutes followed by cooling to 12 °C with a 1.5 °C/s ramp. Plasmid digestion was done using type II restriction enzymes (NEB). The plasmids lenti sgRNA(MS2)_zeo backbone and PB-UniSAM were digested using the enzymes BsmBI and BbsI, respectively. For ligation of gRNAs into linearized plasmids, approximately 60 ng of purified plasmid DNA, 1 µl annealed gRNA oligonucleotides, 1 µl T4 DNA Ligase Reaction Buffer (NEB) and 1 µl T4 DNA Ligase (400 000 units/ml; NEB) were used, and ddH_2_O was added to a total volume of 10 µl. The mixture was incubated in a peqSTAR thermal cycler (Peqlab Biotechnologie) at 16 °C overnight. Resulting recombinant plasmids were designated as [plasmid name + gRNA name #gRNA number].

For preparation of plasmid DNA from *E. coli* liquid cultures, the NucleoSpin Plasmid Kit or the NucleoBond Xtra Maxi Kit were employed (both Macherey-Nagel) and used according to the manufacturer’s protocol. Recombinant plasmids were either sequenced by Seqlab (Göttingen, Germany) or verified via control digestions according to the recommendations of the respective enzyme’s manufacturer.

**Preparation of genomic DNA and RNA, reverse transcription of complementary DNA from RNA**

For preparation of genomic DNA (gDNA), the QIAamp DNA Mini kit (Qiagen, Hilden, Germany) was used according to the manufacturer’s protocol.

For isolation of total RNA, the NucleoSpin RNA kit (Macherey-Nagel) was employed. RNA was eluted in 30 μl RNase-free H_2_O and stored at ‑80 °C. Reverse transcription of complementary DNA (cDNA) from template RNA was done with the Omniscript RT kit (Qiagen), Random Hexamer Primer (0.02 µg/µl) and RiboLock RNase inhibitor (40 units/µl; both Thermo Fisher Scientific). 1 μg RNA was utilized for each reverse transcription.

**Protein isolation and Immunoblot**

Cells were washed with PBS and detached using Trypsin/EDTA. After two washes with PBS, cells were resuspended in radioimmunoprecipitation assay (RIPA) buffer freshly supplemented with protease and phosphatase inhibitors. Cells were lysed by incubation on ice for 35 min with gentle shaking. Cell debris was separated by centrifugation (14 000 *g*, 15 min, 4 °C), and supernatants were stored at ‑80 °C.

Concentrations of protein solutions were determined using the Pierce BCA Protein Assay Kit (Thermo Fisher Scientific) according to the supplier’s instructions. Protein solutions were diluted 1:10 with ddH_2_O for measurement and each sample was analyzed in duplicate using a Tecan Sunrise absorbance reader, provided with Magellan data analysis software (Tecan Group, Männedorf, Switzerland). Samples’ protein concentrations were calculated according to a BSA standard curve.

Protein samples were supplemented with 5x Laemmli buffer on ice, mixed, and incubated at 95 °C for 5 min. The mixture was centrifuged at 11 000 *g* for 2 min and kept on ice until being loaded on an SDS polyacrylamide gel. 5 μl PageRuler Prestained Protein ladder (Thermo Fisher Scientific) was used as a reference for protein sizes. The gel was run at 80 V for initial sample loading and afterwards at 150 V for about 1.5 h in 1x SDS running buffer.

After the run, proteins were transferred from the gel to a Protran BA83 nitrocellulose membrane (Whatman, Dassel, Germany) in a Trans-Blot SD Semi-Dry Transfer Cell (Bio-Rad Laboratories). The system was run at 70 mA for 1 h. After hybridization, the nitrocellulose membrane was stained with Ponceau S solution (Roth) to visualize protein bands and to facilitate cutting of the membrane according to the ladder. The membrane was rinsed with ddH_2_O and was blocked in 1x Tris-buffered saline + 0.1% (v/v) Tween 20 (TBS‑T) supplemented with 5% (w/v) fat-free milk powder for 2 h at RT with gentle shaking.

For immunostaining, horseradish peroxidase (HRP) conjugated primary antibodies were used, each diluted in TBS‑T supplemented with 5% (w/v) fat-free milk powder. A mouse anti-human β‑actin antibody was used in 1:5 000 dilution, whereas a rabbit anti-human NF-κB p65/RelA antibody and a mouse anti-*S. pyogenes* Cas9 antibody were both used in 1:1 000 dilution (listed below). Sections of the membrane were incubated with primary antibodies overnight at 4 °C with gentle shaking and subsequently washed three times with TBS‑T for 10 min. Also secondary antibodies were diluted in TBS‑T supplemented with 5% (w/v) fat-free milk powder. A goat anti-mouse IgG/HRP antibody was used diluted 1:15 000 for detection of β‑actin and diluted 1:2 000 for detection of Cas9. A goat anti-rabbit IgG/HRP antibody was used diluted 1:2 000 for detection of NF-κB p65/RelA (listed below). Respective sections of the membrane were incubated with the corresponding secondary antibodies for 2 h at RT with gentle shaking and again washed three times with TBS‑T.

Detection was done with Amersham ECL Western Blotting Detection Reagent (GE Healthcare Life Sciences) for 5 min in the dark. In a final step, the membrane was exposed to an X-ray film (Amersham Hyperfilm ECL, GE Healthcare Life Sciences) for 5 s ‑ 5 min, which was then developed using an SRX-101A X-ray film processor (Konica Minolta, Tokyo, Japan).

**Lentiviral transduction**

Different approaches were tested for lentiviral transduction. The final protocol was as follows: Pseudotyped lentiviral particles were produced through transfection of HEK‑293T cells. Cell culture supernatants were harvested 48 h and 72 h post transfection, and viral particles were concentrated with Lenti‑X Concentrator (Takara Bio, Kusatsu, Japan). RetroNectin reagent (Takara Bio) was used for plate coating according to the manufacturer’s protocol, except for an additional washing step with PBS before blocking with 2% bovine serum albumin (BSA). At the day of transduction, 5x10^4^ target cells were seeded per 6‑well of RetroNectin-coated plates, and the virus solution was added directly to freshly seeded cells to a total volume of 1 ml serum-free medium. In order to increase transduction efficiency, Polybrene Infection/Transfection Reagent (Merck Millipore, Burlington, USA) was added to a final concentration of 10 µg/ml. Medium was replaced after 3 - 4 h with serum-containing medium, and cells were kept in culture for up to 19 days. Splitting and cell harvests were adapted to the growth rate of the cells in order to keep cells in a subconfluent state constantly. This applied especially to pMSC, as those tend to show more inter-experimental variations regarding proliferation rate and viability after treatment.

Using the SAM system, lentiviral particles transmitting dCas9-VP64, MS2-p65-HSF1 and at least one gRNA have to be combined to achieve a functionally assembled activation system. In order to reduce cell stress, the transduction process was divided into two stages. First, lentiviral particles transmitting the sequences of *dCAS9-VP64* and *MS2-P65-HSF1* were applied in combination as described above. After 2 days, a second transduction was performed with lentiviral particles transmitting the respective gRNAs, which were added to the already seeded and previously transduced target cells. Polybrene addition and medium change were done as before.

**Quantitative real time PCR**

The mix for each quantitative real-time PCR (qRT‑PCR) reaction consisted of 10 µl iTaq Universal SYBR Green Supermix (Bio-Rad Laboratories), 0.6 µl of each F and R primer, 7.8 µl water for molecular biology (Merck Millipore) and 1 µl cDNA. qRT-PCRs were run on an Eppendorf Mastercycler ep gradient realplex^2^ (Eppendorf, Hamburg, Germany), and the program applied was as follows: **1)** initial denaturation at 95 °C for 10 min, **2)** 45 cycles each consisting of denaturation at 94 °C for 15 s, primer annealing at 58 °C for 30 s and elongation at 72 °C for 30 s, and **3)** generation of a melting curve by heating to 95 °C for 10 s, rapid cooling to 58 °C for 15 s and slow heating to 95 °C within 20 min, followed by a final incubation at 95 °C for 15 s. Samples were analyzed in duplicate. Relative quantification of data was done by subtracting expression levels of the reference gene human glyceraldehyde 3‑phosphate dehydrogenase (*GAPDH*) from levels of the target gene for each sample and subsequent normalization to a control sample according to the 2^-ΔΔCt^ method^[5]^. For evaluation and graphical representation of technical replicates, 2^-ΔΔCt^ was calculated individually for each replicate (i.e., CT values were not averaged per sample). Only ΔCt values of the control sample were averaged and subtracted from each individual replicate ΔCt of the other samples.

**Chromatin immunoprecipitation**

For chromatin immunoprecipitation (ChIP), DNA was cross-linked with associated proteins by fixation of living cells, and resulting DNA-protein complexes were sheared to gain smaller fragments. An antibody specific for the protein of interest was used to immunoprecipitate DNA fragments associated with this protein from the pool of different fragments. The amount of specific DNA regions present was determined via quantitative PCR (qPCR). Primers and antibodies were all are listed above. Evaluation was done via qPCR in three technical replicates.

**Analyses with flow cytometry**

For flow cytometric analyses, cells were detached using a Trypsin/EDTA (0.25%/0.02% (w/v)) solution (Biochrom by Merck Millipore, Berlin, Germany) and washed with PBS (centrifugation at 500 *g* for 5 min). Supernatants were discarded, and flow cytometric antibodies were added (see table below). Samples were mixed briefly, incubated for 30 min on ice in the dark, and washed again with PBS. 4′,6-diamidino-2-phenylindole (DAPI) fluorescent stain (AppliChem, Darmstadt, Germany) was added for staining of dead cells, and samples were measured immediately with a BD LSR II or BD FACSAria III flow cytometer (both BD Biosciences) equipped with BD FACSDiva software, version 6.1.3 and 8.02, respectively. Fluorescence of turbo green fluorescent protein (tGFP) or mCherry was measured without additional antibody staining. Cell doublets were excluded via area-width discrimination of either side scatter (SSC) or forward scatter (FSC), and living cells were detected via negative DAPI staining.

Percentages of positive cells for a certain surface antigen were always determined in relation to a negative control. Therefore, a threshold was set to contain 1% positive cells in the control. This threshold was then transferred to all samples, and cells with fluorescence intensities above the threshold were defined as positive for the respective antigen.

**Guide RNA sequences**

| **gRNA name** | **Sequence (5‘ → 3‘ orientation)** | **Application** |
| --- | --- | --- |
| **gRNA_CIITA-pIII #1-F**^a^ | CACCGTGATCACAATGGAACCGCAC | F gRNA targeting pIII of human *CIITA* |
| **gRNA_CIITA-pIII #1-R**^a^ | AAACGTGCGGTTCCATTGTGATCAC | R gRNA targeting pIII of human *CIITA* |
| **gRNA_CIITA-pIII #2-F**^a^ | CACCGTAGCTTGGCGGGCTCCCAAC | F gRNA targeting pIII of human *CIITA* |
| **gRNA_CIITA-pIII #2-R**^a^ | AAACGTTGGGAGCCCGCCAAGCTAC | R gRNA targeting pIII of human *CIITA* |
| **gRNA_CIITA-pIII #3-F**^a^ | CACCGGACTACAAGGGTACCATATT | F gRNA targeting pIII of human *CIITA* |
| **gRNA_CIITA-pIII #3-R**^a^ | AAACAATATGGTACCCTTGTAGTCC | R gRNA targeting pIII of human *CIITA* |
| **gRNA_CIITA-pIV #1-F**^b^ | CACCGACCACGTGCTTTATCAGAAG | F gRNA targeting pIV of human *CIITA* |
| **gRNA_CIITA-pIV #1-R**^b^ | AAACCTTCTGATAAAGCACGTGGTC | R gRNA targeting pIV of human *CIITA* |
| **gRNA_CIITA-pIV #2-F**^b^ | CACCGGGCCACTGTGAGGAACCGAC | F gRNA targeting pIV of human *CIITA* |
| **gRNA_CIITA-pIV #2-R**^b^ | AAACGTCGGTTCCTCACAGTGGCCC | R gRNA targeting pIV of human *CIITA* |
| **gRNA_CIITA-pIV #3-F**^b^ | CACCGGAGCAACCAAGCACCTACTG | F gRNA targeting pIV of human *CIITA* |
| **gRNA_CIITA-pIV #3-R**^b^ | AAACCAGTAGGTGCTTGGTTGCTCC | R gRNA targeting pIV of human *CIITA* |
| **gRNA_CEBPA #1-F**^c^ | CACCGGGCTCTGCTGGGCGCGCTGG | F gRNA targeting the promoter of human *CEBPA* |
| **gRNA_CEBPA #1-R**^c^ | AAACCCAGCGCGCCCAGCAGAGCCC | R gRNA targeting the promoter of human *CEBPA* |
| **gRNA_CEBPA #2-F**^c^ | CACCGAGCGCGGCGCTGTGCCGCGC | F gRNA targeting the promoter of human *CEBPA* |
| **gRNA_CEBPA #2-R**^c^ | AAACGCGCGGCACAGCGCCGCGCTC | R gRNA targeting the promoter of human *CEBPA* |
| **gRNA_CEBPA #3-F**^c^ | CACCGGCCGCGCGCTAGGACCCAGC | F gRNA targeting the promoter of human *CEBPA* |
| **gRNA_CEBPA #3-R**^c^ | AAACGCTGGGTCCTAGCGCGCGGCC | R gRNA targeting the promoter of human *CEBPA* |
| **gRNA_Control-F**^c^ | CACCGGTCTTCGGAGGACAGTACTC | F control gRNA  (no target) |
| **gRNA_Control-R**^c^ | AAACGAGTACTGTCCTCCGAAGACC | R control gRNA  (no target) |

^a^	Designed with crispr.mit.edu online design tool by Feng Zhang lab (Massachusetts Institute of Technology, Cambridge, USA)

^b^	Designed with Custom Alt-R CRISPR-Cas9 guide RNA design tool by Integrated DNA Technologies (Coralville, USA)

^c^	Furuhata *et al.*, 2017

**Primer sequences**

| **Primer name** | **Sequence**  **(5‘ → 3‘ orientation)** | **Application** |
| --- | --- | --- |
| **Taq *GAPDH* 1** | CTCCTCCACCTTTGACGCTG | F qRT-PCR primer for amplifi­cation of human *GAPDH* from cDNA (house-keeping gene) |
| **Taq *GAPDH* 2** | ACCACCCTGTTGCTGTAGCC | R qRT-PCR primer for amplifi­cation of human *GAPDH* from cDNA (house-keeping gene) |
| **q*CIITA*-3-F** | TTATGCCAATATCGCGGAACTG | F qRT-PCR primer for amplifi­cation of human *CIITA* from cDNA |
| **q*CIITA*-3-R** | CATCTGGTCCTATGTGCTTGAAA | R qRT-PCR primer for amplifi­cation of human *CIITA* from cDNA |
| ***qHLA-DR*alpha-F** | TCTGGCGGCTTGAAGAATTTG | F qRT-PCR primer for amplifi­cation of human *HLA-DR* alpha chain from cDNA |
| ***qHLA-DR*alpha-R** | GGTGATCGGAGTATAGTTGGAGC | R qRT-PCR primer for amplifi­cation of human *HLA-DR* alpha chain from cDNA |
| **q*CEBPA*-F**^a^ | AACACGAAGCACGATCAGTC | F qRT-PCR primer for amplifi­cation of human *CEBPA* from cDNA |
| **q*CEBPA*-R**^a^ | AGACGCGCACATTCACATTG | R qRT-PCR primer for amplifi­cation of human *CEBPA* from cDNA |
| **dCas9-F2**^b^ | CCAAAGAGGTGCTGGACG | F primer used for amplification of the insert of lenti dCAS-VP64_Blast from gDNA/cDNA |
| **Blast-R**^b^ | GCTCTTTCAATGAGGGTGGA | R primer used for amplification of the insert of lenti dCAS-VP64_Blast from gDNA/cDNA |
| **Hygro-F**^b^ | GACGGCAATTTCGATGATG | F primer used for amplification of the insert of lenti MS2-P65-HSF1_Hygro from gDNA/cDNA |
| **pBluescript-KS**^b^ | TCGAGGTCGACGGTATC | R primer used for amplification of the insert of lenti MS2-P65-HSF1_Hygro from gDNA/cDNA |
| **h*CIITA*-pI-F** | TGGGCTTCTCTGGCACAT | F primer used for ChIP-qPCR of pI of human *CIITA* |
| **h*CIITA*-pI-R** | GGCTTTTGGGTTGATTCAGA | R primer used for ChIP-qPCR of pI of human *CIITA* |
| **h*CIITA*-pII-F** | CCTTCCCAATTCCAAGAGGT | F primer used for ChIP-qPCR of pII of human *CIITA* |
| **h*CIITA*-pII-R** | GTCCCTGAGCCCTTAGATCAG | R primer used for ChIP-qPCR of pII of human *CIITA* |
| **h*CIITA*-pIII-F** | AGAGGGTGTTCAGAAACAGAAATC | F primer used for ChIP-qPCR of pIII of human *CIITA* |
| **h*CIITA*-pIII-R** | AAGCTAAGCCAACATGCAAAG | R primer used for ChIP-qPCR of pIII of human *CIITA* |
| **h*CIITA*-pIV-F** | GTTGGGATGCCACTTCTGAT | F primer used for ChIP-qPCR of pIV of human *CIITA* |
| **h*CIITA*-pIV-R** | CTGTGGAGCAACCAAGCAC | R primer used for ChIP-qPCR of pIV of human *CIITA* |

^a^	Furuhata *et al.*, 2017

^b^	Addgene (Watertown, USA)

**Plasmids**

| **Plasmid name** | **Description** | **Reference** |
| --- | --- | --- |
| psPAX2 | Lentiviral packaging plasmid encoding viral capsid and enzymes | Didier Trono;  Addgene #12260 |
| pMD2.G | Lentiviral packaging plasmid encoding viral receptor protein | Didier Trono;  Addgene #12259 |
| lenti dCAS-VP64_Blast | Lentiviral plasmid encoding dCas9-VP64 fusion protein | Konermann *et al.*, 2015; Addgene #61425 |
| lenti MS2-P65-HSF1_Hygro | Lentiviral plasmid encoding MS2-p65-HSF1 activator helper complex | Konermann *et al.*, 2015; Addgene #61426 |
| lenti sgRNA(MS2)_zeo backbone | Lentiviral backbone plasmid for gRNA cloning | Konermann *et al.*, 2015; Addgene #61427 |
| pGIPZ | Lentiviral backbone plasmid encoding TurboGFP from *Pontellina plumata* | Dharmacon, GE Healthcare, Lafayette, USA |
| PB-UniSAM | *PiggyBac* transposon plasmid for gRNA cloning; encoding dCas9-VP64, MS2-p65-HSF1 and mCherry fluorescent protein | Fidanza *et al.*, 2017; Addgene #99866 |
| pCMV-hyPBase | Expression plasmid of hyperactive *piggyBac* transposase hyPBase | Yusa *et al.*, 2011 |

**Antibodies**

| **Antibody name** | **Clone** | **Application** | **Supplier** |
| --- | --- | --- | --- |
| PerCP anti-human HLA‑DR | L243 | Flow cytometry | BioLegend, San Diego, USA |
| IgG from rabbit serum | - | Chromatin immuno-precipitation (ChIP) | Sigma-Aldrich |
| purified anti-human RNAPII subunit RPB1 CTD | 8WG16 | Chromatin immuno-precipitation (ChIP) | BioLegend |
| anti-H3K4me3 (pAb-003-050) | polyclonal | Chromatin immuno-precipitation (ChIP) | Diagenode, Liège, Belgium |
| anti-H3K27ac (C15410174) | polyclonal | Chromatin immuno-precipitation (ChIP) | Diagenode |
| mouse anti-human β-actin (A1978) | AC-15 | Western Blot | Sigma-Aldrich |
| rabbit anti-human NF-κB p65/RelA (#8242) | D14E12 | Western Blot | Cell Signaling Technology, Danvers, USA |
| mouse anti-*S. pyogenes* Cas9 (C15200216) | 4G10 | Western Blot | Diagenode |
| goat anti-mouse IgG/HRP | polyclonal | Western Blot | Dako, Glostrup, Denmark |
| goat anti-rabbit IgG/HRP | polyclonal | Western Blot | Dako |

**Gene Identification**

| **Gene (homo sapiens)** | **Gene ID (NIH)** | **Gene ID (Ensembl)** |
| --- | --- | --- |
| GAPDH | 2597 | [ENSG00000111640](http://www.ensembl.org/id/ENSG00000111640) |
| CEBPa | 1050 | ENSG00000245848 |
| CIITA | 4261 | [ENSG00000179583](http://www.ensembl.org/id/ENSG00000179583) |
| HLA-DRa | 3122 | [ENSG00000204287](http://www.ensembl.org/id/ENSG00000204287) |
| ACTINb | 60 | ENSG00000075624 |

**Statistical analyses and software**

The crispr.mit.edu online design tool (Feng Zhang lab, Massachusetts Institute of Technology) and the Custom Alt‑R CRISPR-Cas9 guide RNA design tool (Integrated DNA Technologies) were applied for gRNA design. Target sequence specificity of all gRNAs and primers was verified using the Primer-BLAST online tool^[6]^. Analyses of qRT PCR data were done with Excel 365 (Microsoft, Redmond, USA). Flow cytometry data were evaluated using FlowJo v10.6.1 software (FlowJo, BD Life Sciences). Statistical analyses and graphical representation of data were done using GraphPad Prism 10.2.3 and 8.1.1 (GraphPad Software, San Diego, USA). Final assembly of figures was done with PowerPoint 2016 (Microsoft, Redmond, USA).

**Supplementary Tables**

**Supplementary Table S1: Approaches tested for establishment of lentiviral transduction and CRISPR/Cas9 SAM in pMSC**

| **Method** | **Description** | **Results** |
| --- | --- | --- |
| RetroNectin* | Coating of dishes | Improved growth of MSC |
| Freezing of viral supernatants | Short-time storage of viral supernatants at -80 °C | Reduced transduction efficiency |
| Spinofection | Non-concentrated viral supernatant was added and the plates were centrifuged at 4 000 rpm for 20 min at 32 °C. The procedure was repeated five times with fresh viral supernatant each. Finally, target cells were added together with fresh viral supernatant and centrifuged at 2 000 rpm for 1 h at 32 °C. | MSC became detached and died |
| Lenti-X concentrator* | Concentration of viral supernatants | Higher amounts of viral particles could be added per well without the need for spinofection |
| Varied incubation time of target cells with viral particles | Different durations from 1 h to overnight were tested | Incubation for 3 - 4 h appeared to be a good compromise between effective transduction and minimized cell stress* |
| Protamine sulfate | Transduction enhancer; final concentrations of 5 - 100 µg/ml were tested | No target gene enhancement observed, whereas MSC survival was impacted at higher doses |
| Polybrene* | Transduction enhancer; final concentration of 10 µg/ml (1 - 10 µg/ml were tested) | Transduction enhanced and viability retained |
| Valproic acid (VPA) | Histone deacetylase inhibitor; final concentration of 1 mM | No enhanced expression of target genes in pMSC |
| Decitabine | DNA methyltransferase inhibitor final concentration of 3.5 µM | No enhanced expression of target genes in pMSC |
| PB-UniSAM + pCMV-hyPBase (alternative to lentiviral transduction) | *PiggyBac* transposon plasmid for gRNA cloning; transfection with calcium phosphate | Transfection and target gene activation was achieved in HEK-293T cells but not in pMSC  (Supplementary Figure S8) |
| PB-UniSAM + pCMV-hyPBase (alternative to lentiviral transduction) | *PiggyBac* transposon plasmid for gRNA cloning; nucleofection with the Amaxa™ Nucleofector II device | Most MSC died |

* These approaches were kept throughout the following procedures

**Supplementary Figures**

**Supplementary Figure S1**

**Fig. S1: Verification of SAM components in gDNA and protein.** In order to confirm the uptake, integration and processing of the SAM components in HEK-293T and pMSC after transfection/transduction, **A** gDNA was extracted and PCR analysis was performed, followed by agarose gel electrophoresis. Untreated cells and H_2_O served as negative controls and plasmids were used as positive controls. Positive PCR product signals in pMSC were visible at 460 bp length for dCas9-VP64 and 831 bp for MS2-P65-HSF1. A 100 bp DNA ladder was used for comparison of band sizes. **B** In order to verify successful translation, immunoblotting was done with transfected / transduced cells. Again, samples from untreated HEK‑293T and pMSC were used as negative controls. Expected band sizes were 165 kDa for dCas9-VP64, 100<130 kDa for MS2-p65-HSF1, 65 kDa for wild type p65 and 42 kDa for β‑actin. All components were visible in HEK-293T cells in high amounts, and in pMSC, the MS2-p65-HSF1protein was clearly detectable. PageRuler Prestained Protein ladder (Thermo Fisher Scientific) was used for comparison of protein sizes. Green marks on the X-ray film indicate the ladder and the cutting edges of the membrane.

**Supplementary Figure S2**

**Fig. S2: *CEBPA* gene expression determined with qRT-PCR for three gRNAs.**

qRT-PCR analysis with *GAPDH* as control gene; expression depicted as fold-change compared to cells transduced with a non-targeting control gRNA: **A** In transfected HEK-293T cells, 7‑fold and 5‑fold fold induction of *CEBPA* was achieved with gRNA_CEBPA #1 and #3. Pooled application of all three gRNAs resulted in a 13‑fold induction. **B** In transduced T47D cells, gRNA_CEBPA #1, #3 and the gRNA pool induced *CEBPA* 6‑fold, 16-fold, and 25-fold, respectively. **C** In transduced pMSC, gene expression was induced 3‑fold using gRNA_CEBPA #3, 14-fold with gRNA_CEBPA #1 and 14-fold with the gRNA pool. Single gRNA_CEBPA #2 did not induce *CEBPA* in HEK-293T, T47D and pMSC. Error bars represent standard deviation of two technical replicates.

**Supplementary Figure S3**

**Fig. S3: Induction of *CIITA* and *HLA-DR* after treatment with IFN‑γ.**

Gene expression levels were determined with qRT-PCR analysis normalized to the *GAPDH* control gene. **A** After treatment of pMSC with IFN‑γ at concentrations of 20 ‑ 100 units/ml for 2 days, target gene expression levels were determined in relation to those of untreated control cells. Maximum of 108‑fold up-regulation for *CIITA* and 60‑fold for *HLA‑DR* was achieved with 100 U/ml IFN‑γ. **B** In PBMC, the IFN‑γ-induced activation of *CIITA* and *HLA-DR* was less then 2-fold. **C** A comparative time course experiment with 100 U/ml IFN‑γ exposure between 1h and 24h was carried out in pMSC, PBMC and T47D cells. *CIITA* expression of PBMC was hardly affected by IFN‑γ, but in pMSC and T47D cells, the levels of *CIITA* expression started after 1 h and peaked after 4 h of treatment: 65‑fold in MSC and 85‑fold in T47D. **D** Flow cytometric analysis of pMSC after IFN‑γ treatment for 2 h and for 5 days revealed an enhancement of mean fluorescence intensity (MFI) of the fluorophore-conjugated anti-HLA‑DR antibody with a 8‑fold induction after 2h and a 18‑fold induction after 5 days compared to untreated control cells. Quantification of the primary data shown in Supplementary Figure S9. Error bars represent standard deviation of two technical replicates.

**Supplementary Figure S4**

**Fig. S4: Basal gene expression of *CIITA* and *HLA-DR* in MSC and PBMC.** Gene expression levels were determined via qRT-PCR analysis. *GAPDH* served as reference gene. In direct comparison between pMSC and PBMC, the basal *CIITA* expression level was 664-fold higher and the basal *HLA‑DR* expression level even 1 064-fold higher in PBMC. Error bars represent standard deviation of two technical replicates.

**Supplementary Figure S5**

**Fig. S5: Induction of *CIITA* and *HLA-DR* in HEK-293T and T47D cells.** Gene expression levels of *CIITA* and *HLA-DR* after transfection or transduction were determined via qRT-PCR analyses with *GAPDH* as control gene. **A** After transfection of HEK‑293T cells with the CRISPR/Cas9-SAM, *CIITA* expression was induced 66‑fold and *HLA‑DR* expression 39‑fold with gRNA_CIITA‑pIII #2, and *CIITA* 5-fold with gRNA_CIITA‑pIII #1. For gRNA_CIITA‑pIII #3, no activation of *CIITA* but of *HLA‑*DR was observed (3‑fold). The gRNA pool led to a 131‑fold and 110‑fold activation of *CIITA* and *HLA‑DR* gene expression, respectively. **B** Flow cytometric analysis of HLA-DR protein on HEK‑293T cells revealed HLA‑DR surface protein in 9.9% and 6.7% of cells transfected with gRNA_CIITA‑pIII #2 and the gRNA pool, respectively. Quantification of the primary data shown in Supplementary Figure S6. **C** Lentiviral transduction for overexpression of *CIITA* via pIII in T47D cells with gRNA_CIITA‑pIII #2 induced *CIITA* 4-fold and *HLA‑DR* 18-fold. **D** For overexpression of *CIITA* via pIV in HEK-293T cells, 32/33-fold up-regulation of *CIITA* and 17/14‑fold induction of *HLA-DR* was observed with gRNA_CIITA‑pIV #2/#3. With gRNA_CIITA‑pIV #1, *CIITA* was induced 9‑fold and *HLA‑DR* 6-fold. With the gRNA pool, *CIITA* was induced 93‑fold and *HLA-DR* 27‑fold. **E** In T47D cells, gRNA_CIITA‑pIV #2 induced *CIITA* and *HLA‑DR* gene expression 104‑fold and 363‑fold. gRNA_CIITA‑pIV #3 induced *CIITA* 32‑fold and *HLA-DR* 250‑fold. *CIITA* expression was induced 85‑fold and *HLA‑DR* expression 326‑fold by the gRNA pool. **F** Flow cytometric analysis of T47D cells transduced with the SAM components and gRNA_CIITA‑pIV #2 revealed 85% of HLA‑DR positive cells. Error bars represent standard deviation of two technical replicates. Different symbols in **C** indicate biological replicates.

**Supplementary Figure S6**

**Fig. S6: HLA-DR protein expression in HEK-293T cells with gRNAs targeting pIII**. After transfection with the SAM system and gRNA_CIITA‑pIII #1, #2, #3 and pool (#1-2-3), surface protein expression of HLA-DR on HEK-293T cells was measured with flow cytometric analysis. Untreated cells served as control (NC) and the percentages of positive cells were measured in relation to the fluorescence signal of the control cells.

**Supplementary Figure S7**

**Fig. S7: Transduction with the SAM system and gRNA_CIITA-pIII #2 and concomitant treatment with VPA/Decitabine/DMSO in pMSC**. In order to test for enhancement of promotor recruitment, pMSC were treated with 1 mM valproic acid (VPA), 3.5 µM decitabine and DMSO as solvent control, but qRT-PCR analysis with *GAPDH* as reference gene revealed only minimal changes of *CIITA* or *HLA-DR* gene expression levels. Error bars represent standard deviation of two biological replicates.

**Supplementary Figure S8**

**Fig. S8: Application of PB-UniSAM in pMSC and HEK-293T cells.** As an alternative to lentiviral transduction, PB‑UniSAM was equipped with gRNA_CIITA‑pIII #2 and was applied in combination with the transfer inducer plasmid pCMV-hyPBase. **A** In HEK-293T cells, *CIITA* and *HLA-DR* were inducible (i.e., 15‑fold activation of *CIITA* and 4‑fold induction of *HLA-DR*), but no induction was observed in pMSC. Error bars represent standard deviation of two technical replicates. **B** Fluorescence of the plasmid’s mCherry marker was measured after transfection and a turboGFP-encoding plasmid was used as a control. While 99% of HEK-293T cells were found positive for turboGFP and 22% positive for mCherry, expression of both fluorescence markers could not be detected in pMSC.

**Supplementary Figure S9**

**Fig. S9: Primary data of HLA-DR surface expression measurement employing flow cytometric analysis after pMSC treatment with IFN-γ.** IFN-γ treatment was performed for 2 hours and 5 days. The fluorescence intensity of treated pMSC as well as untreated control cells is depicted.

1. Nold P, Brendel C, Neubauer A, Bein G, & Hackstein H. Good manufacturing practice-compliant animal-free expansion of human bone marrow derived mesenchymal stroma cells in a closed hollow-fiber-based bioreactor. Biochemical and biophysical research communications 2013;430(1):325-330.
2. Burk J, Holland H, Lauermann AF, May T, Siedlaczek P, Charwat V, et al. Generation and characterization of a functional human adipose-derived multipotent mesenchymal stromal cell line. Biotechnol Bioeng. 2019;116(6):1417-26.
3. Fidanza A, Lopez-Yrigoyen M, Romano N, et al. An all-in-one UniSam vector system for efficient gene activation. Sci Rep. 2017;7(1):6394.
4. Ye J, Coulouris G, Zaretskaya I, Cutcutache I, Rozen S, Madden TL. Primer-BLAST: a tool to design target-specific primers for polymerase chain reaction. BMC Bioinformatics. 2012 Jun 18;13:134.
5. Livak KJ, Schmittgen TD. Analysis of relative gene expression data using real-time quantitative PCR and the 2(-Delta Delta C(T)) Method. Methods. 2001;25(4):402-408. doi.org/10.1006/meth.2001.1262.
6. Ye J, Coulouris G, Zaretskaya I, et al. Primer-BLAST: a tool to design target-specific primers for polymerase chain reaction. BMC Bioinformatics. 2012;13:134. doi.org/10.1186/1471-2105-13-134.
